# Household transmission dynamics of COVID-19 among residents of Delhi, India: a prospective case-ascertained study

**DOI:** 10.1016/j.ijregi.2023.02.005

**Published:** 2023-02-23

**Authors:** Farzana Islam, Yasir Alvi, Mohammad Ahmad, Faheem Ahmed, Anisur Rahman, Farishta Hannah D. Singh, Ayan Kumar Das, Mridu Dudeja, Ekta Gupta, Rashmi Agarwalla, Iqbal Alam, Sushovan Roy

**Affiliations:** a Department of Community Medicine, Hamdard Institute of Medical Science and Research, New Delhi, India; b World Health Organization, Country Office, India; c Department of Public Health, King Khalid University, Abha, Kingdom of Saudi Arabia; d Department of Microbiology, Hamdard Institute of Medical Science and Research, New Delhi, India; e Scientist-E, National Institute of Cancer Prevention and Research, ICMR, Noida, India; f Department of Community and Family Medicine, All India Institute of Medical Science, Guwahati, India; g Department of Physiology, Hamdard Institute of Medical Science and Research, New Delhi, India

**Keywords:** COVID-19, secondary attack rate, community spread, epidemiological characteristics, close-contact transmission

## Abstract

**Objective:**

The aim of this study was to observe the secondary infection rate and transmission dynamics of COVID-19 among household contacts, and their associations with various factors across four dimensions of interaction.

**Methods:**

This was a case-ascertained study among unvaccinated household contacts of a laboratory-confirmed COVID-19 case in New Delhi between December 2020 and July 2021. For this study, 99 index cases and their 316 household contacts were interviewed and sampled (blood and oro-nasal swab) on days 1, 7, 14, and 28.

**Results:**

The secondary infection rate among unvaccinated household contacts was 44.6% (95% confidence interval (CI) 39.1–50.1). The predictors of secondary infection among individual contact levels were: being female (odds ratio (OR) 2.13), increasing age (OR 1.01), symptoms at baseline (OR 3.39), and symptoms during follow-up (OR 3.18). Among index cases, age of the primary case (OR 1.03) and symptoms during follow-up (OR 6.29) were significantly associated with secondary infection. Among household-level and contact patterns, having more rooms (OR 4.44) and taking care of the index case (OR 2.02) were significantly associated with secondary infection.

**Conclusion:**

A high secondary infection rate highlights the need to adopt strict measures and advocate COVID-19-appropriate behaviors. A targeted approach for higher-risk household contacts would efficiently limit infections among susceptible contacts.

## Highlights

• Household settings give a clearer picture of infectious disease transmission dynamics.
• Four dimensions of risk factors for understanding secondary infection are proposed.
• A high secondary attack rate highlights the need for COVID-appropriate behaviours.
• A targeted approach could be adopted in limiting disease among household contacts.
• Our four-dimensional approach to understanding household transmission is relevant.

## Graphical Abstract

**Figure:**
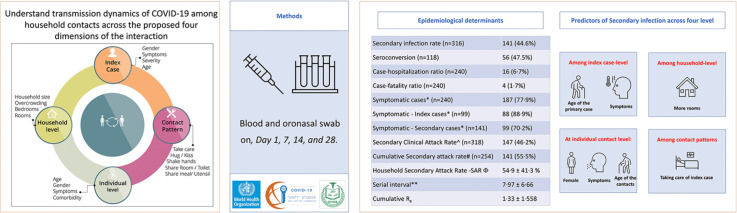

## Introduction

The COVID-19 pandemic has been one of the most widespread and devastating disasters humanity has witnessed this century. The uncertainty over its epidemiological behavior has made disease control even more challenging. With the world left paralyzed for almost 2 years, the resumption of normalcy will depend upon how well we understand this virus's epidemiology and transmissibility patterns. As closed settings, households are crucial components in spreading the respiratory disease, having also played a dominant role in earlier pandemics involving swine flu (H1N1) and severe acute respiratory syndrome (SARS) [1,2]. The literature shows varied secondary infection rates of SARS-CoV-2 in household contacts, ranging from 4.6% to 50% [3].

Household-based studies provide an enumerable set of people exposed to an infectious person, and are therefore valuable in assessing the key transmission dynamics of SARS-CoV-2 [4]. Especially during lockdowns, with limited interactions among community members and different households, suspected contacts are in a closed setting, presenting an opportunity to observe infectious disease transmission patterns. Thus, household settings allow determination of the virus's transmission dynamics and help us to understand the clinical spectrum of illness in secondary cases [2]. Overcrowding in households can increase the risk of transmission, supposedly leading to decreased effectiveness of lockdown and stay-at-home measures in low- and middle-income countries (LMICs), where providing a quality healthcare service for the entire population has been a challenge [5].

**Figure 1:**
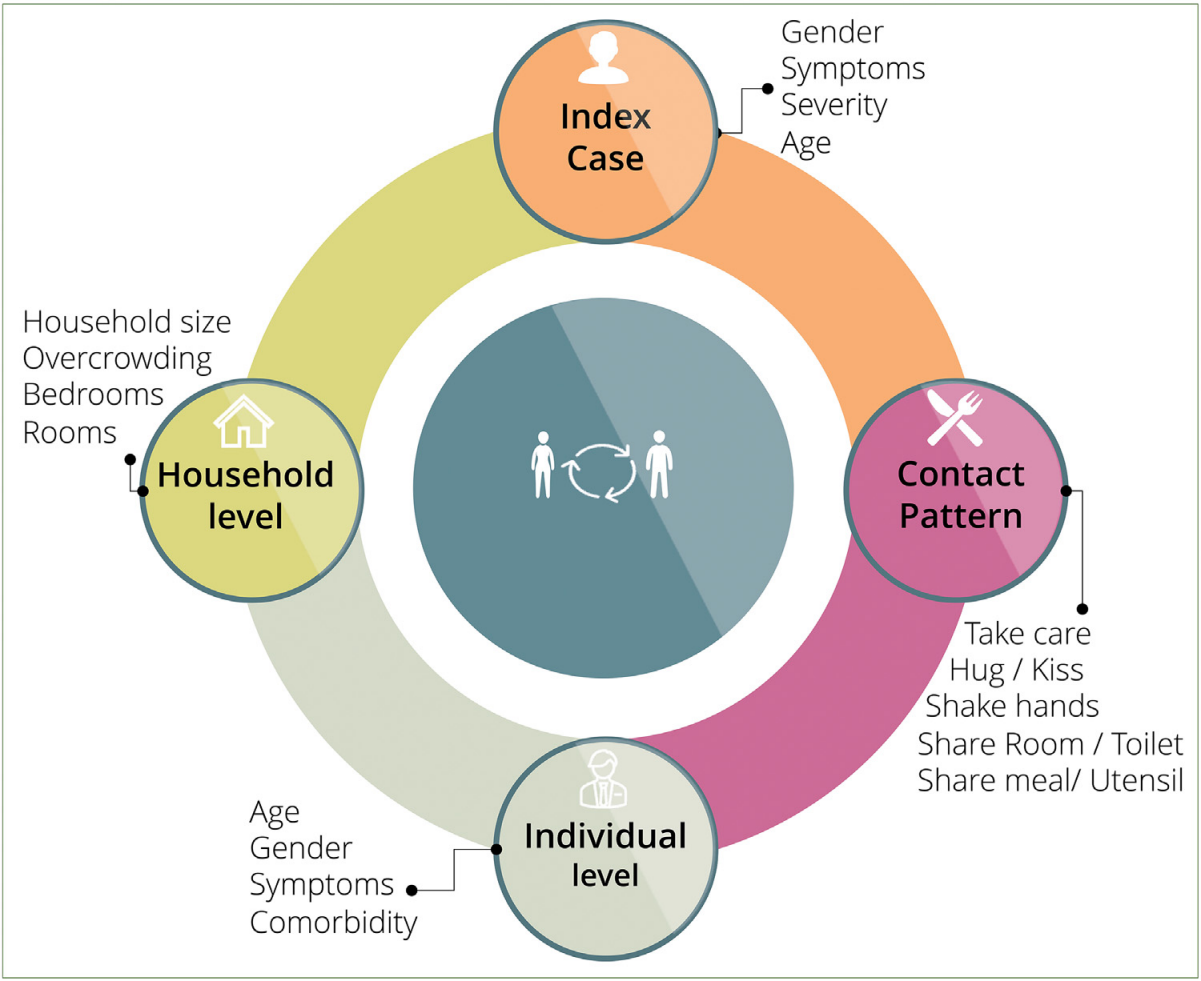
Factors governing transmission dynamics among household members across proposed four dimensions. Disease transmission among household members is influenced by interplay of four sets of factors, including index case characteristics (age, gender, presence of symptoms and their severity), household level (household size, overcrowding, number of bedrooms, room for isolation), individual level (age, gender, presence of symptoms, any comorbidity), and contact pattern (interaction with index case in taking care, hugging, kissing, handshaking, sharing room and toilet, sharing meals and utensils).

In early 2021, India had the world's second-highest number of COVID-19 cases [6,7]. India's capital, New Delhi, witnessed two sharp peaks in 2020 and 2021, with the second wave being one of the deadliest, potentially linked to the new B.1.617.2 ‘Delta’ variant's highly contagious nature [7]. The dynamics of transmission of COVID-19 infection among household contacts remain unclear. Evidence suggests that COVID-19 transmission among household contacts depends on several factors [8]. Our study conceptualized this phenomenon as being governed by the interplay of four sets of factors: index case characteristics, household level, individual level, and contact patterns (Figure 1). Investigating the extent of infection among household contacts can provide helpful information on virus transmissibility and routes of transmission. Moreover, such studies can also be used to assess the reliability of our conceived four-dimensional approach to understanding COVID-19 transmission dynamics among household contacts. Thus, the aim of our study was to observe secondary infection rates and examine the transmission dynamics of COVID-19 among household contacts, and their associations with various factors across four dimensions of interaction.

## Methods

Our study was based on WHO UNITY protocols, developed to guide the investigation of the transmission dynamics and clinical spectrum of COVID-19 infection, by generating standardized, high-quality, and comparable studies [9].

### Study setting and population

This was a prospective case-ascertained study conducted among unvaccinated household contacts of laboratory-confirmed COVID-19 patients residing in Delhi's South and South-East districts. The study was conducted from December 2020 to June 2021, covering India's first and second waves of the COVID-19 pandemic. The first wave hit India in March 2020 and continued until December 2020, peaking in mid-September, while the second, deadlier wave was observed from March to July 2021 [6]. During this period, whenever a new COVID-19 positive patient was detected, contact tracing and surveillance were performed by the district health authority for 14 days, and all the suspected contacts with symptoms were advised to undergo testing, although no mandate existed.

The inclusion criteria were:

• households with a confirmed COVID-19 case identified, with at least one other household member;
• all contacts of the confirmed COVID-19 case residing in the same household;
• residing in the South and South-East districts of Delhi.

The exclusion criteria were:

• date of COVID-19 symptom onset same for more than one household member;
• cases and contacts who were hospitalized on day 1.
• individuals residing in hostel accommodation.

### Operational definitions

**Figure 2:**
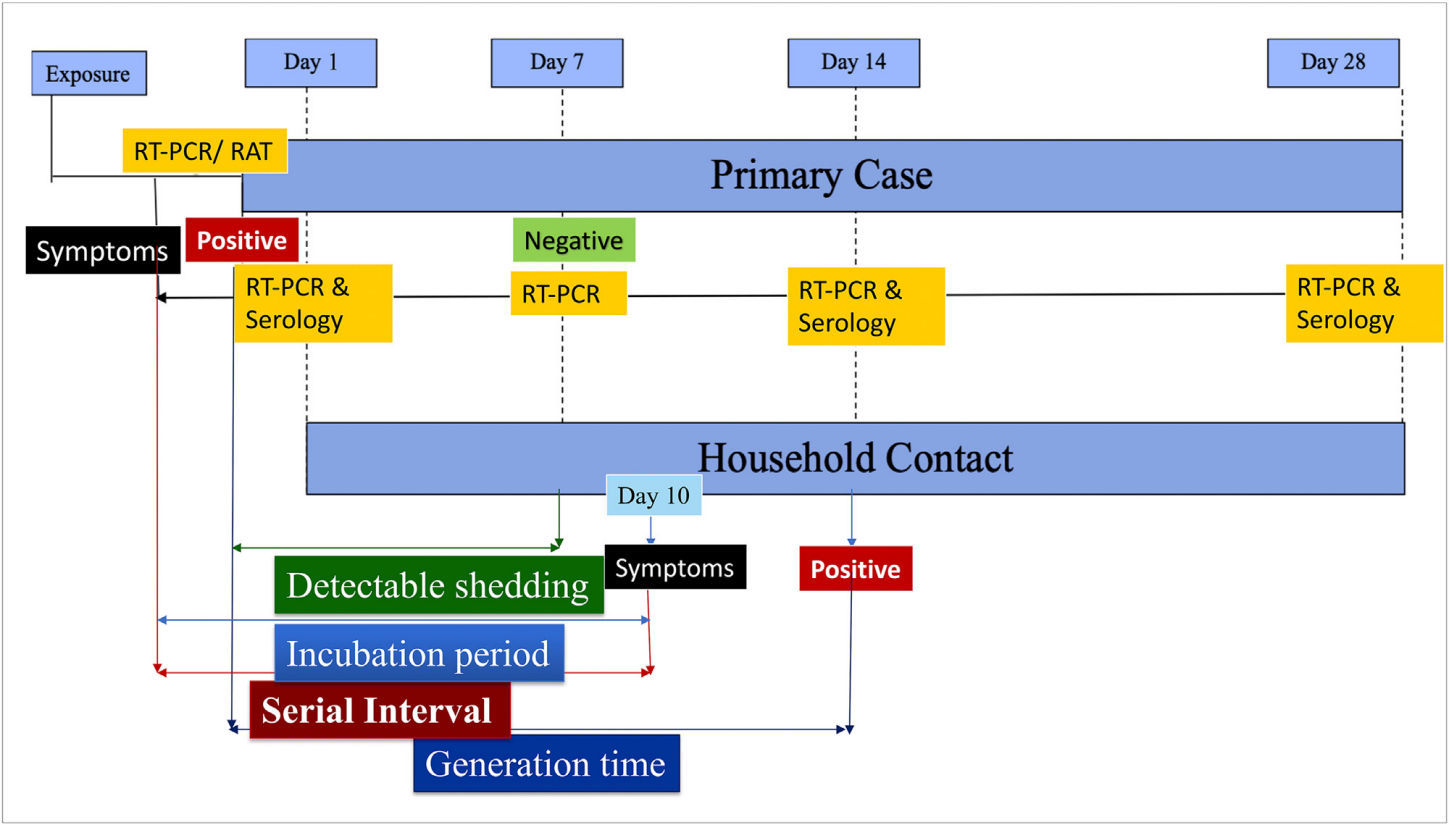
Illustration of household-level transmission of COVID -19 infection in our study population. Exposure to the primary case in the household is the first event followed by symptoms in him/her and a subsequent positive test for COVID-19. In our illustration, we enrolled primary case on Day 1, shortly after he/she tested positive for COVID -19. We assumed that by Day 7 the primary case would have shredded all of the viruses and the PCR testing would be negative. Exposed household contacts may experience symptoms during the second week, which can be used to calculate the incubation period (time from exposure to onset of symptoms in contacts) and the serial interval (time from onset of symptoms in the primary case to onset in contact). The primary case would subsequently test positive for COVID-19 on Day 14 assessment and can be used to calculate the generation time (time between testing positive of primary case and contact).

The following definitions were based on the WHO UNITY protocol (Figure 2) [9]:

• *Secondary case/infection* — those contacts who were enrolled *and* confirmed for new infections of COVID-19, assessed using reverse transcription polymerase chain reaction (RT-PCR), anytime within 28 days of follow-up.
• *Secondary infection rate* — the proportion of household contacts confirmed positive for COVID-19, assessed using RT-PCR anytime within 28 days of follow-up, divided by total enrolled contacts.
• *Seroconversion* — those contacts confirmed for new infections of COVID-19, assessed using serological assays on paired samples, with a change from initial negative to positive anytime within 28 days of follow-up.
• *Secondary clinical attack rate* — the proportion of household contacts with symptoms suggestive of COVID-19 (fever, sore throat, runny nose, cough, shortness of breath, loss of smell or taste, muscle ache, headache) within 14 days of the index case testing positive, divided by total enrolled contacts. This cut-off was based on the district health authority's contact tracing period of 14 days, to allow comparisons between Indian studies.
• *Secondary attack rate (cumulative SAR)* — the proportion of household contacts positive for COVID-19, assessed using RT-PCR within 14 days from the index case testing positive, divided by all contacts tested.
• *Household Secondary Attack Rate (SAR)* — calculated at the household level, this was the proportion of enrolled household contacts positive for COVID-19, assessed through RT-PCR within 14 days from the index case testing positive, divided by all contacts tested.
• *Serial interval* — the period from the onset of symptoms in the primary case to the onset of symptoms in a susceptible household contact, irrespective of their infection status.
• *Detectable shedding* — the time required for the RT-PCR positive case (index and secondary) to become negative.
• *Effective reproduction number, R_e_* — the average number of infections produced per index case in their household.

### Sample size

Considering a secondary attack rate of 0.5 at the household level, a prevalence of 50% [10], [11], [12], [13] was assumed. Using 90% power and a 95% confidence interval, and applying the Schwartz formula, the sample size came out to be 384, which was rounded up to 400 household contacts. Assuming an average family size of five, it was decided to include 100 households in our study. After identification of the index case, all eligible contacts in that household were enrolled. Those not consenting to participate in the study were excluded.

### Study tools

The study tools comprised:

• baseline questionnaire
• follow-up questionnaire
• symptom diary
• samples (nasopharyngeal and oropharyngeal swab, and blood serum).

Face-to-face interviews with the cases (or family members if the patient was too ill to be interviewed) and household members were carried out to collect the necessary information, using baseline and follow-up questionnaires. The data collection team comprised trained healthcare workers and a phlebotomist who visited the family at their house. All precautionary measures, according to government guidelines, were followed during data collection. Baseline data and samples were collected on the day of recruitment (day 1), while follow-up data were collected during home visits on days 7, 14, and 28. COVID-19 was confirmed using molecular RT-PCR, and serology was performed to identify baseline antibody status and seroconversion (Supplementary Figure 1). All household contacts and index cases were provided a symptom diary to record their symptoms for 28 days.

### Laboratory analysis

Nasopharyngeal and oropharyngeal (respiratory) samples were collected on days 1, 7, 14, and 28, and serum samples on days 1, 14, and 28 from all the study participants (cases and contacts). Respiratory samples were analyzed by RT-PCR according to government guidelines. Serology was performed as per government guidelines and testing strategies, utilizing WANTAI kits provided by WHO (see Supplementary Material).

### Enrollment strategy

The study was carried out in the COVID-19 testing center of Hamdard Institute of Medical Science and Research (HIMSR), New Delhi, along with other testing centers. A list of patients who tested positive at HIMSR was prepared daily, and the first two patients giving consent were included in the study. However, due to the decline in the number of cases, deviation from the study protocol did occur. Next, a list of COVID-19 positive patients from two districts (Delhi's South and South-East districts) was assessed for enrollment [protocol deviation 1]. Retrospective enrolments were then performed if there were insufficient enrollments in one particular week [protocol deviation 1]. After the initial visit (day 1), subsequent follow-up visits were conducted on days 7, 14, and 28. For the retrospective enrolments, the first visit was scheduled on day 7 (from the index case testing positive).

### Statistical analysis

The data were entered in MS Excel and analyzed using IBM Statistical Package for Social Sciences (SPSS) version 26. The study datasets are available from the data depository as open access [14]. The categorical variables and proportions were represented by percentage (%), while continuous variables were described using the mean and standard deviation or median with interquartile range, wherever applicable. Associations between various factors with the primary outcome as secondary infection were assessed by logistic regression. All variables, with *p* < 0.1 in univariable logistic regression, were further analyzed using multivariable logistic regression via the enter method. Probability (*p*) was based on a 5% level of significance.

### Ethical considerations

This study was based on the WHO UNITY protocol. It was approved by the WHO Research Ethics Review Committee (ERC.0003356) and the Institutional Ethical Committee of HIMSR, New Delhi (IEC-2020/15). The manuscript was further approved by the Publications Review Committee WHO-HQ (PRC-21-120603). Approvals were also obtained from the district authority, hospital, and medical college. Informed written consent was obtained from every participant after explaining the study's intended purpose, confidentiality, autonomy, and beneficence. Health education and referral, if required, were given appropriately.

## Results

**Figure 3:**
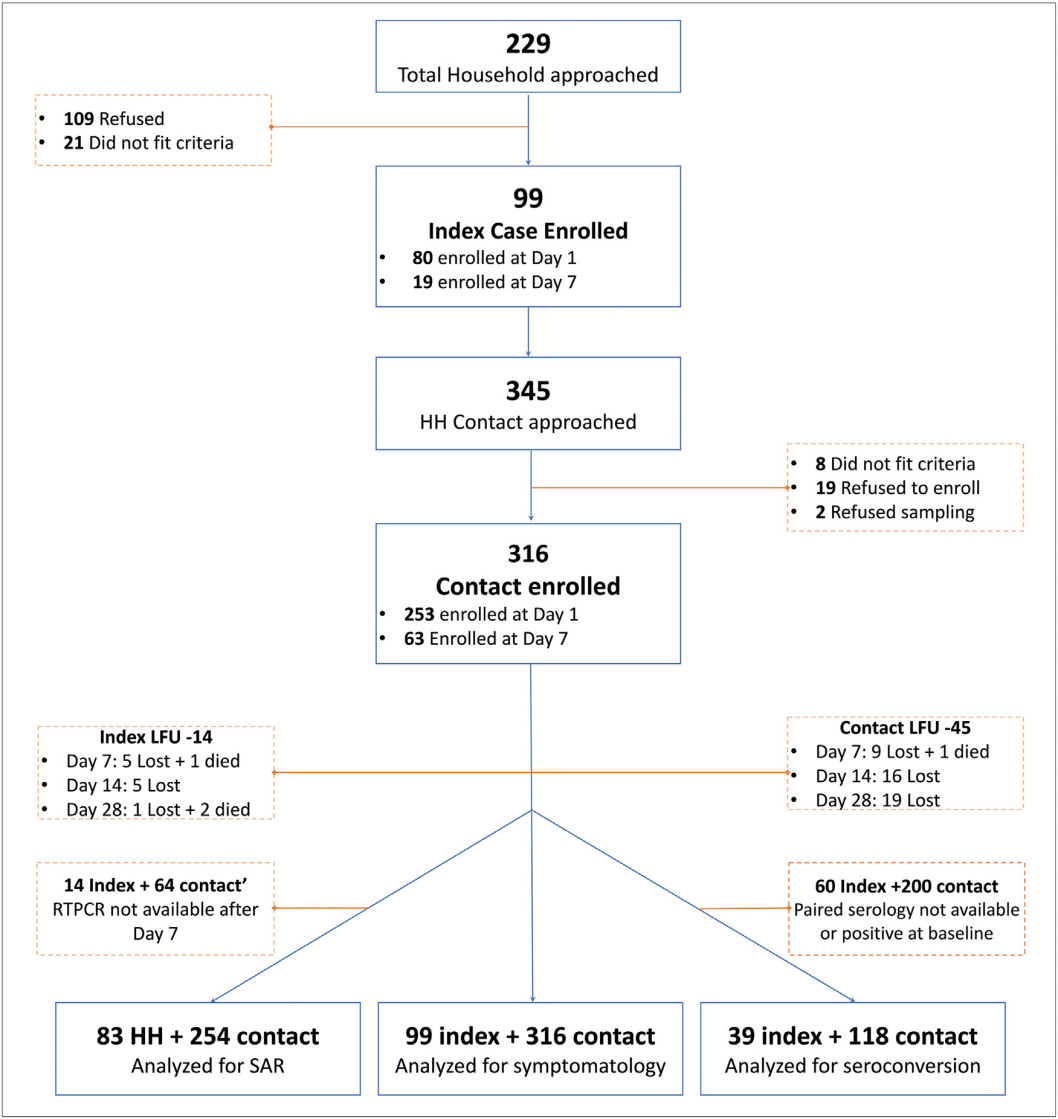
Flow diagram describing study enrolments and participant eligibility for analyses.

In total, 99 index cases and their 316 household contacts were enrolled in this study. Details of the enrollment process are given in Figure 3. Table 1 shows the participants’ sociodemographic data. Household characteristics are presented in Supplementary Table 1, and the types of contact involved in Supplementary Table 2. As shown in Table 1, there was an equal gender distribution among household contacts; however, more of the primary cases were male. The mean age was 38.7 ± 14.4 years among primary cases, and 33.3 ± 19.4 years among the contacts. The mean household size was 4.5 ± 2.0, while the median number of rooms in the households was three. At the time of enrollment, 84.8% (95% confidence interval (CI) 76.4–90.7) of index cases were symptomatic, while 29.7% (CI 25.0–35.0) of household contacts also reported symptoms. However, the proportion of symptomatic cases and contacts decreased with each follow-up (Supplementary Table 3). Supplementary Figure 2 shows the symptom profiles of index cases, and Supplementary Figure 3 shows the comorbidity profiles of index cases and household contacts.

**Table 1:** Sociodemographic profile of study participants.

Characteristics		Primary cases (*N* = 99)	Household contacts (*N* = 316)	Secondary cases (*N* = 141)
**Gender**	Male	59 (59.6%)	154 (48.7%)	54 (38.3%)
	Female	40 (40.4%)	162 (51.3%)	87 (61.7%)
Age*	Mean (SD)	38.7 ± 14.38	33.2 ± 19.4	36.0 ± 20.0
	Median (IQR)	37 (28–47)	30 (17–48.50)	35 (22–50)
Age groups*	< 15	2 (2.1%)	63 (19.9%)	23 (16.3%)
	15–49	73 (73.6%)	175 (55.4%)	82 (58.2%)
	50–59	13 (13.1%)	44 (13.9%)	15 (10.6%)
	60–69	10 (10.1%)	21 (6.6%)	10 (7.1%)
	≥ 70	1 (1.1%)	13 (4.1%)	11 (7.8%)
**Occupation**#	Healthcare worker	16 (16.2%)	16 (5.1%)	9 (6.4%)
	Government sector	5 (5.1%)	11 (3.5%)	5 (3.5%)
	Private sector	16 (16.1%)	56 (17.6%)	25 (17.7%)
	Business/self-employed	22 (22.2%)	39 (12.3%)	13 (9.2%)
	Homemaker/stay at home	26 (26.3%)	92 (29.1%)	46 (32.6%)
	Retired	7 (7.1%)	10 (3.2%)	9 (6.4%)
	Student	7 (7.1%)	89 (28.2%)	34 (24.1%)
	Unemployed	0 (0%)	3 (0.9%)	0 (0%)

⁎ In years

# Self-reported

**Table 2:** Household contacts’ PCR, serology, and symptoms during the follow-up period.

Follow-up day	RT-PCR positive *n* (%*)	Serology positive *n* (%*)	Symptoms present *n* (%@)
Day 1	167 (66.5%)	85 (40.3%)	94 (29.7%)
Day 7	83 (32.3%)	22 (36.1%)	107 (33.9%)
Day 14	27 (13.4%)	71 (60.2%)	66 (20.9%)
Day 28	6 (3.5%)	91 (61.1%)	15 (4.7%)

*n*: frequency

⁎ Among those who were tested

@ Among total household contacts (*N* = 316)

Table 3 shows the transmission dynamics of COVID-19 among household contacts. The secondary infection rate among household contacts was 44.6% (CI 39.1–50.1). Across all COVID-19 cases, 6.7% (CI 4.1–10.6) were hospitalized, and the case fatality rate was found to be 1.7% (CI 0.6–4.2). The proportion of cases with symptoms was 77.9% (CI 72.3–82.7). The clinical attack rate was observed to be 46.5% (41.1–52.0), while the secondary attack rate was 55.5% (CI 49.4–61.6). Other transmission dynamics, including serial interval, detectable shedding, and effective reproduction number, are also given in Table 3.

**Table 3:** Transmission dynamics among household contacts.

**Secondary infection rate (*n* = 316)**		141 (44.6%)
**Seroconversion (*n* = 118)**		56 (47.5%)
**Case-hospitalization ratio (*n* = 240)**		16 (6.7%)
**Case-fatality ratio (*n* = 240)**		4 (1.7%)
Symptomatic cases*(*n* = 240)		187 (77.9%)
Symptomatic — index cases*(*n* = 99)		88 (88.9%)
Symptomatic — secondary cases*(*n* = 141)		99 (70.2%)
**Secondary clinical attack rate**^**(*n* = 316)**		147 (46.5%)
**Cumulative secondary attack rate**#**(*n* = 254)**		141 (55.5%)
**Household secondary attack rate**Φ**(*n* = 83)**	Mean (SD)	54.9 ± 41.3 %
	Median (IQR)	57.1 (0 - 100) %
**Serial interval**€	Mean (SD)	7.97 ± 6.66
	Median (IQR)	6.0 (3.0-10.0)
**Detectable shedding among index cases**§	Mean (SD)	12.49 ± 6.78
	Median (IQR)	14 (7-14)
**Detectable shedding among household contacts**§	Mean (SD)	11.84 ± 6.475
	Median (IQR)	7 (7-14)
**Cumulative *R*_e_**	Mean (SD)	1.33 ± 1.558
	Median (IQR)	1.0 (0.0 – 2.0)

*n*: frequency*, R*_e_: number of secondary cases per household

⁎ Any time during 4-week follow-up

^ Contacts showing any symptoms within 14 days from index case testing positive

# Contacts testing positive for COVID-19 within 14 days from index case testing positive among total study population

Φ Contacts testing positive for COVID-19 within 14 days from index case testing positive at household level

€ Time in days between the onset of symptoms in the primary case to the onset of symptoms in a susceptible household contact

§ Time in days for RT-PCR positive cases to become negative

**Table 4:** Predictors of secondary infection among household contacts.

Predictor		Secondary infection		OR (95% CI)	*p*-value	Adjusted OR (95% CI)	*p*-value
		Yes (*N* = 141) *n* (%)	No (*N* = 175) n (%)				
**1. Individual contact-level characteristics**							
**Gender**	Male	54 (35.1%)	100 (64.9%)	1		1	
	Female	87 (53.7%)	75 (46.3%)	2.15 (1.37–3.38)	0.001	2.13 (1.27–3.57)	0.004
Age*	Mean	36.0 ± 19.9	31.1 ± 18.7	1.01 (1.00–1.02)	0.026	1.01 (1.00–1.03)	0.048
**Symptoms at baseline**	No	68 (30.6%)	154 (69.4%)	1			
	Yes	73 (77.7%)	21 (22.3%)	7.87 (4.48–13.82)	< 0.001	3.39 (1.61–7.12)	0.001
**Symptom follow-up**	No	42 (24.9%)	127 (75.1%)	1		1	
	Yes	99 (67.3%)	48 (32.7%)	6.23 (3.81–10.18)	< 0.001	3.18 (1.64–6.19)	0.001
**Any comorbidity**#	No	122 (44.0%)	155 (56.0%)	1			
	Yes	19 (48.7%)	20 (51.3%)	1.20 (0.61–2.36)	0.58		
**2. Index case-level characteristics**							
**Gender**	Male	96 (49.5%)	98 (50.5%)	1			
	Female	45 (36.9%)	77 (63.1%)	2.14 (1.36–3.37)	< 0.001	0.67 (0.41–1.10)	0.113
Age*	Mean (SD)	43.56 ± 15.39	35.15 ± 15.64	1.03 (1.02–1.05)	< 0.001	1.03 (1.01–1.04)	0.001
**Symptoms**	No	6 (13.6%)	38 (86.4%)	1			
	Yes	135 (49.6%)	137 (50.4%)	5.58 (2.53–12.31)	< 0.001	6.29 (1.83–21.63)	0.003
**3. Household-level characteristics**							
**Household size**^	< 5	60 (44.4%)	75 (55.6%)	1.10 (0.63–1.93)	0.72		
	5–6	47 (47.0%)	53 (53.0%)	1.22 (0.67–2.21)	0.50		
	> 7	34 (42.0%)	47 (58.0%)	1			
**Rooms**	1–2	13 (19.7%)	53 (80.3%)	1			
	3–4	78 (53.4%)	68 (46.6%)	4.68 (2.35–9.31)	< 0.001	4.44 (2.16–9.13)	< 0.001
	> 5	50 (48.1%)	54 (51.9%)	3.78 (1.84–7.74)	< 0.001	3.67 (1.77–7.59)	< 0.001
**Overcrowding**Φ	Yes	57 (38.8%)	90 (61.2%)	0.64 (0.41–1.003)	0.052	0.89 (0.55–1.45)	0.638
	No	84 (49.7%)	85 (50.3%)	1		1	
**4. Contact pattern**€							
**Share room**	No	54 (44.3%)	68 (55.7%)	1			
	Yes	87 (44.8%)	107 (55.2%)	1.02 (0.64–1.61)	0.919		
**Take care**	No	80 (38.1%)	130 (61.9%)	1			
	Yes	61 (57.5%)	45 (42.5%)	2.20 (1.36–3.54)	0.001	2.02 (1.21–3.38)	0.007
**Hug**	No	122 (43.1%)	161 (56.9%)	1			
	Yes	19 (57.6%)	14 (42.4%)	1.79 (0.86–3.71)	0.117		
**Kiss**	No	135 (44.9%)	166 (55.1%)	1			
	Yes	6 (40.0%)	9 (60.0%)	0.82 (0.28–2.36)	0.713		
**Shake hands**	No	125 (43.4%)	163 (56.6%)	1			
	Yes	16 (57.1%)	12 (42.9%)	1.73 (0.79–3.80)	0.167		
**Share meal**	No	96 (41.6%)	135 (58.4%)	1			
	Yes	45 (52.9%)	40 (47.1%)	1.58 (0.96–2.60)	0.072	1.16 (0.61–2.21)	0.645
**Share plate**	No	137 (45.4%)	165 (54.6%)	1			
	Yes	4 (28.6%)	10 (71.4%)	0.48 (0.14– 1.57)	0.226		
**Share cup**	No	137 (45.4%)	165 (54.6%)	1			
	Yes	4 (28.6%)	10 (71.4%)	0.48 (0.14– 1.57)	0.226		
**Share utensil**	No	129 (44.0%)	164 (56.0%)	1			
	Yes	12 (52.2%)	11 (47.8%)	1.38 (0.59–3.24)	0.451		
**Sleep in the same room**	No	115 (43.9%)	147 (56.1%)	1			
	Yes	26 (48.1%)	28 (51.9%)	1.18 (0.66–2.13)	0.567		
Share toilet	No	84 (40.8%)	122 (59.2%)	1			
	Yes	57 (51.8%)	53 (48.2%)	1.56 (0.98–2.48)	0.061	1.12 (0.61–2.05)	0.720

OR: odds ratio, CI: confidence interval, *n*: frequency

⁎ In years

^ Number of members in the household

# Including diabetes, hypertension, obesity, cancer, heart disease, asthma, thyroid disease, liver disorder

Φ More than two persons per room

€ Self-reported

The predictors of infection were analyzed across the proposed four dimensions. Among the contact-level characteristics, female contacts had higher odds of testing positive (odds ratio (OR) 2.13, CI 1.27–3.57). Similarly, increasing age (OR 1.01, CI 1.00–1.03), presence of symptoms at baseline (OR 3.39, CI 1.61–7.12), and having symptoms during the follow-up (OR 3.18, CI 1.64–6.19) were found to be significant predictors of COVID-19 infection among the household contacts. Among the index case-level characteristics, increasing age (OR 1.03, CI 1.01–1.04) and presence of symptoms (OR 6.29, CI 1.83–21.63) led to higher odds of household contacts testing positive, after adjusting for gender. Among the household-level characteristics, more rooms in the house (OR 3.67, CI 1.77–7.59) was a significant predictor of COVID-19 infection among the contacts, while household size and overcrowding were not significantly associated. Among contact patterns, those household members who took care of the index cases had higher odds of testing positive (OR 2.02, 1.21–3.38), while the effects of other contact patterns were insignificant.

## Discussion

The aim of this study was to improve the understanding of the transmission dynamics of COVID-19 among household contacts of index cases, along with clinical and epidemiological characteristics of the infection. Our four dimensions of interaction in determining secondary infections among household contacts were also assessed.

More males were observed among the index cases, whereas females outnumbered males in the secondary cases. Female household members were more likely to test positive for COVID-19 than males. This was also observed in a cohort study from China, where females had a higher risk (RR = 1.6) than males [15]. In India, it is usually women who care for sick family members, increasing their risk of contracting the infection. In contrast, researchers from Spain found that gender had no effect on disease transmission among contacts [16]. Other studies have also found no association between gender and secondary infection, but have found spouses of the index case to be at a higher risk [3,[17], [18], [19], [20]. Increasing age among household contacts and index cases was also observed to be a significant and independent predictor of secondary infection among household members. Researchers around the world have documented similar findings [15], [16], [17], although some studies failed to record any significant relationship between age and secondary infection [18], [19]. In China, Zhang et al. studied changes in the pattern of transmission dynamics and concluded that younger individuals had a lower risk of infection than older individuals [13].

The households in our study had an average of 4.4 members, and overcrowding was observed in 33% of families. Since COVID-19 is an infectious disease with high infectivity, families with large household sizes were expected to have higher secondary infection rates. However, our results showed lower secondary infection rates in households with increasing household size and overcrowding, although this association was not significant. A contrasting finding was observed with respect to the number of rooms, where significantly higher rates of secondary infection were found in households with more rooms. Thompson et al. found a trend of decreasing transmissibility with increasing household size, while Wang et al. observed that households with fewer contacts had higher rates of secondary infection [21], [22]. Similar trends have been observed in studies done around the world, with a higher secondary attack rate (SAR) among small households compared with larger ones [16], [17], [18].

With regard to the dimension of index–contact interaction, the household members were more likely to be infected through contact with the index case [23]. In our study, more than half of the members shared a room, a third of them gave care, and a third shared a toilet. Those contacts who cared for the index case had a higher risk of contracting infection, while other contact-level interactions were not significant. The findings from one systematic review suggested that transmission among contacts was influenced by group interactions, including sharing meals or playing board games [24]. A study conducted in Hangzhou, China found that the risk of secondary infection was 31.6 times among family members and 2.6 times among those who dined together [25]. Many of the index–contact interactions found to be significant by other research did not show similar associations in our study. Early isolation and COVID-appropriate behavior, which was made mandatory by the district administration in the study population, could be one reason. Since the households in our study had low rates of overcrowding and sufficient rooms for isolation, interaction with index cases was limited. However, it was not possible to compare data on different types of contact among the household members, which could have a bearing on secondary infections.

Regarding symptomatology, the primary cases showed more symptoms, with longer duration, than household contacts and secondary cases. Among household contacts who later tested positive (secondary cases), two-thirds showed symptoms, mostly in the first week, with one-third of these also symptomatic in the second week. Fever and sore throat were the major complaints, followed by cough and muscle aches. Other studies have observed fever, cough, and sore throat to be the most common clinical presentations [26], [27]. The less common symptoms among our study participants were myalgia, fatigue, nausea, and vomiting — similar to studies carried out elsewhere [22,[26], [27], [28]. Our study found more secondary infections among households in which the primary case was symptomatic (adjusted OR (aOR): 3.4). This finding supports the idea that symptomatic index cases transmit COVID-19 infections to their contacts much more than asymptomatic cases [21]. This concurred with the findings of Madewell et al., in which the secondary attack rate (SAR) from symptomatic index cases was 18%, compared with 0.7% for asymptomatic cases [17]. Other reviews and meta-analyses have observed similar findings, i.e. a higher SAR among symptomatic index cases than for asymptomatic cases [3,[16], [17],[19], [20],[29], [30].

The majority of the household contacts tested COVID-19 positive in the first week, with positive serology reaching a maximum at the final follow-up. The case-hospitalization and the case-fatality ratios were 6.7% and 1.7%, respectively. The national figures for these ratios were lower, although studies among specific populations have reported similar or even higher rates [31]. These rates were also affected by the epidemic wave of diseases as well as circulating virus variants. Thus, a more detailed, trend-wise distribution of hospitalization and death among the enrolled cases, along with genotyping, would have been more helpful in understanding household transmission and the severity of the disease. The serial interval was 8 days, comparable to that found in China, which ranged from 5.5 to 7.5 days [22,32].

Among the transmission dynamics factors, secondary attack rate (SAR) was also calculated —cumulative as well as at the household level; the cumulative SAR was 55.5%, while the household figure was 54.9%. These rates were very high compared with the results of some previous studies, ranging from 9.3% in southern India and 11.1% in Spain to 16–34% in China and 25% in Japan [5,^18^,^26^,28]. Pooled SARs from a meta-analysis were observed to vary between 7% and 21.1% [17,[20], [21],33]. All of these rates are much lower than those observed in our study. However, other studies have observed SARs to be similar or even higher. Similar rates were documented in Finland (48%), Thailand (48%), and China (54.9%) [11], [12], [13],34]. In the initial phase of the epidemic, researchers from Taiwan observed a high SAR of 84%, while a small case series from south India reported a rate of 60% [10,35].

These discrepancies need to be understood, for example in terms of methodological differences, especially with regard to the enrollment of contacts. Our study actively ascertained the household contacts, irrespective of their symptoms and/or higher risks. In contrast, studies passively observing attack rates among contacts presenting for testing may have underestimated SARs. Furthermore, our study was conducted during India's deadliest wave of COVID-19, with Delhi facing the most significant impact, resulting in a high SAR. A meta-regression analysis conducted by Tian et al. on studies measuring SARs in different settings indicated that the household and social gathering settings accounted for elevated rates [33]. These differences in SAR could also be attributed to structural and virological differences between the viruses. The lower severity of SARS-CoV-2 could have promoted household transmission, since most cases were kept under home isolation [17]. Since SAR depends not merely on the causative organism, but also on other factors, such as environmental and sociodemographic variables, such heterogeneity is to be expected [20]. One interesting finding was that, despite a high SAR, our results produced an effective reproduction number (*R*_e_) of 1.33 ± 1.56, which was lower in comparison with previous studies (*R*_0_ = 3.38) [34]. This can be explained by the lower number of susceptible individuals among household contacts, since many were seropositive at the baseline. This supports the concept proposed by Yang Liu — that a higher SAR with a low *R*_0_ implies that only a tiny proportion of high-risk contacts were the driving force behind household-level transmission [36]. In our study, the women who were the primary caregivers of the index case were at the highest risk of contracting the infection among the household members. This finding shows that having a targeted high-risk approach could enable us to reduce both SAR and *R*_e._

Our study had some limitations. Firstly, the convenience sampling to enroll index cases, reasonable refusals, and losses to follow-up during the four household visits could have resulted in selection bias and affected the generalization of the findings. Secondly, due to the daily household interactions, it was not possible to ascertain the exact date of exposure to the index case required to calculate any epidemiological characteristics with precision. Furthermore, some definitions were based on symptoms nonspecific to COVID-19, which may have overestimated the outcomes. At the time of this study, there were half a dozen COVID-19 variants; therefore, genotyping studies would have helped to understand transmission patterns. Finally, while the sample size for estimating SAR was calculated, it may not have been sufficient to find the predictors, even though there were more than 20 samples for each independent variable to regress. As this study was a part of the WHO UNITY studies program, pooling epidemiological data from other institutes globally would have helped to reduce these limitations to some extent.

## Conclusion

The secondary attack rate in our setting was very high, highlighting the need to adopt strict measures and COVID-19 appropriate behaviors. Our multidimensional approach to understanding household transmission was shown to be relevant, with significant relationships between secondary infections and many factors. Given the importance of individual-level and index-case characteristics, an approach targeted at household contacts with higher risk would be more efficient in limiting the development of infections among susceptible contacts.

## CRediT authorship contribution statement

**Farzana Islam:** Data curation, Funding acquisition, Formal analysis, Methodology, Project administration, Supervision, Writing – review & editing. **Yasir Alvi:** Data curation, Formal analysis, Methodology, Supervision, Writing – review & editing. **Mohammad Ahmad:** Conceptualization, Funding acquisition, Methodology, Supervision, Validation, Writing – review & editing. **Faheem Ahmed:** Conceptualization, Funding acquisition, Methodology, Supervision, Validation, Writing – review & editing. **Anisur Rahman:** Conceptualization, Funding acquisition, Methodology, Supervision, Validation, Writing – review & editing. **Farishta Hannah D. Singh:** Formal analysis, Writing – review & editing. **Ayan Kumar Das:** Data curation, Formal analysis, Writing – review & editing. **Mridu Dudeja:** Project administration, Supervision, Writing – review & editing. **Ekta Gupta:** Data curation, Methodology, Writing – review & editing. **Rashmi Agarwalla:** Data curation, Methodology, Writing – review & editing. **Iqbal Alam:** Conceptualization, Project administration, Supervision, Writing – review & editing. **Sushovan Roy:** Conceptualization, Project administration, Supervision, Writing – review & editing.

## Conflicts of interest

The authors have declared that no competing interests exist.

## Appendix Supplementary materials

Image, application 1
