# Supplementary material for: Household transmission dynamics of COVID-19 among residents of Delhi, India: a prospective case-ascertained study
Source: IJID Reg. 2023 Feb 23;7:22–30. doi: 10.1016/j.ijregi.2023.02.005 (PMC9946776; doi:10.1016/j.ijregi.2023.02.005)
Supplement: Supplementary file 1 [file mmc1.docx]

**Title: Household Transmission Dynamics of COVID-19 among Residents of Delhi, India: A Prospective Case-ascertained Study**

**METHODS**

**Operational Definitions**

Case of COVID-19: A person with laboratory confirmation of COVID-19 infection by Reverse Transcriptase Polymerase Chain Reaction (RT-PCR), irrespective of clinical signs and symptoms.

Household contact: Any person who has resided in the same household (or other closed settings) as a confirmed COVID-19 case.

**Study plan:**

The details of COVID-19 positive patients were collected from the institute’s lab on the same day of testing (Day 1) as well as from the list that was given every day by the district authority. Initially, the address and phone no. of the positive patient was noted and they were contacted telephonically and after explaining the purpose of the study, verbal consent was taken and a visit to their household was conducted on the same day for Day 1 data and microbiological collection. After visiting the household, detailed written consent was taken from cases as well as from the household contacts.

For cases, data was collected using Form 1A for the first visit, followed by Forms 2, 3 and 4. For contacts, data was collected using Form 1B for the first visit, followed by Forms 2, 3 and 4 (Supplementary Figure 1).

**Specimen collection and transport**

All baseline respiratory (nasopharyngeal and oropharyngeal swab) and serum samples were collected from confirmed cases and their household contacts as per GOI India’s guidelines including any persons without symptoms who have been screened and found to be positive for COVID-19, as soon as possible after laboratory confirmation. Respiratory samples were collected on days 1,7,14 and 28. Paired blood sera for antibody titre were collected on Day 1 (baseline and no later than 7 days after symptom onset for cases and exposure with the confirmed case for contacts) and follow-up sample at least 14 days after the baseline sample or 28 days after symptom onset for cases and last exposure for contacts. Appropriate personal protective equipment (PPE) was worn when specimens were being collected from confirmed cases^.^

All the staff involved in collecting and transporting specimens were trained in safe handling practices and spill decontamination procedures. For each biological sample collected, the time of collection, the conditions for transportation and the time of arrival at the study laboratory were recorded and all efforts were done to transport the specimens to the laboratory as soon as possible after collection.


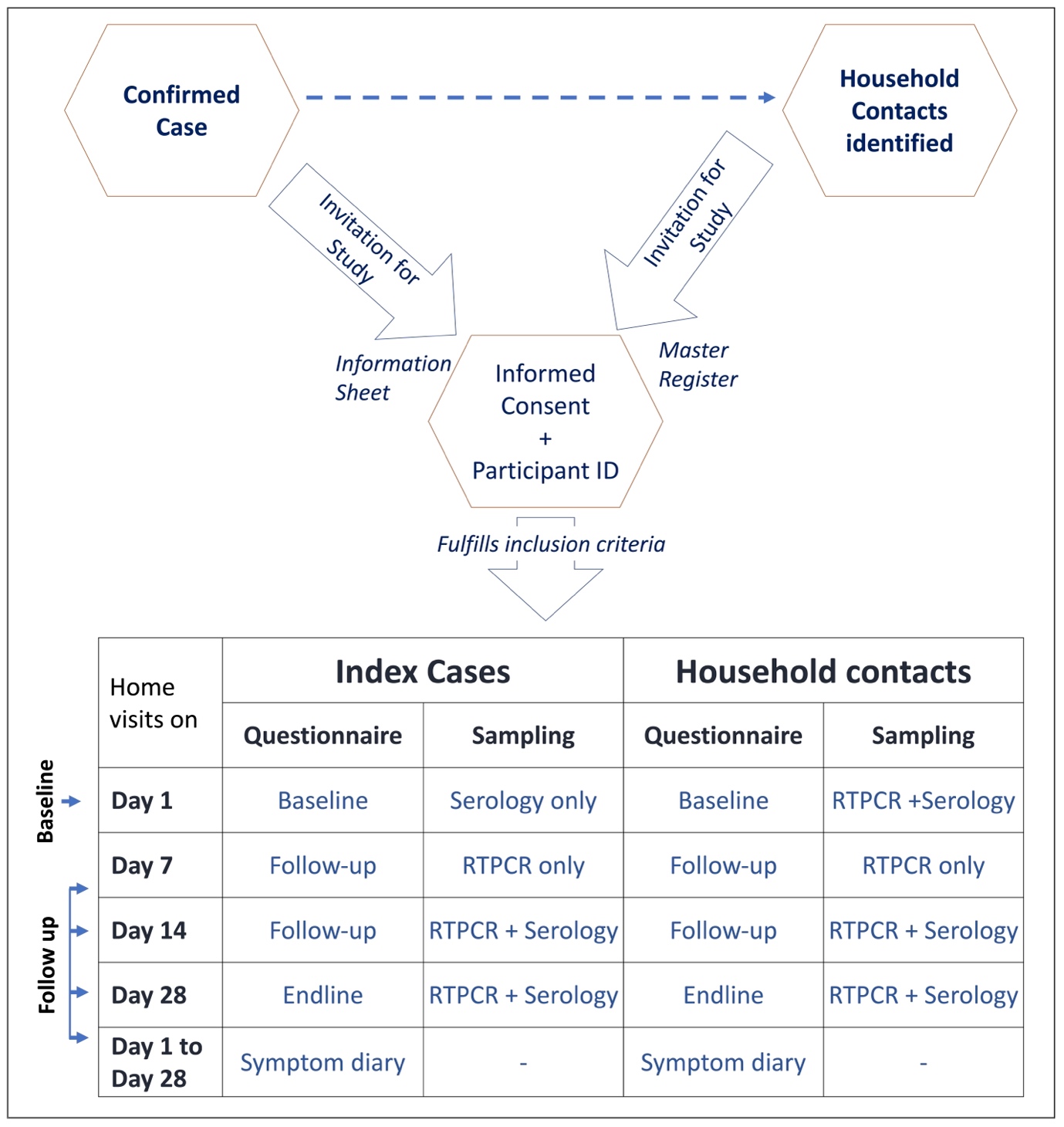


**Supplementary Figure 1: Flowchart of data collection tools and follow up**

**Sample receipt and processing at the testing laboratory:**

Samples were first checked at the testing lab for appropriate labelling, correct test request, sample adequacy, absence of leakage and hemolysis before accepting them. The received samples were then stored appropriately till the initiation of the testing process.

**Detection of SARS COV2 RNA:**

SARS COV2 RNA was detected in the nasopharyngeal and oropharyngeal (NP/OP) swab collected from the subjects by RT PCR following guidelines laid down by ICMR. The swabs collected in viral transport medium (VTM) were processed at the Biosafety level 2 molecular biology laboratory following all the safety protocols. The Viral RNA was extracted from the VTM using QIAamp Viral RNA mini kit, Qiagen, USA, following the kit protocol. Extracted RNA was further analyzed on the same day and was subjected to RT PCR using COVIWOK RT PCR Kit, SNP technologies, Turkey. The kit detects the N gene and the RdRp Gene of SARS COV2. Both the extraction and RT PCR kit are approved by ICMR. The assay was run in the Agilent MX3005P RT PCR system. The CT value cut off for both the viral genes and the internal control was considered as < 35, as mandated by ICMR. The results were communicated and uploaded into the ICMR portal. (20) The samples were stored for future reference for the next 6 months at -80^0^C.

**Anti-SARS-CoV-2-total antibody detection:** Anti-SARS-CoV-2-total antibody was detected in patient samples using Wantai SARS-CoV-2-Ab ELISA kit. The kit detects total antibodies against the SARS-CoV-2 virus and is based on the principle of two-step incubation antigen “sandwich” enzyme immunoassay. Briefly, 100 ml of the patient’s serum is added to polystyrene microwell strips pre-coated with recombinant SARS-CoV-2 antigen. Three wells are marked as negative calibrator and 2 wells as positive calibrator. 50 ml of negative and positive calibrator are added to respective wells and the plate is incubated at 37^0^C for 30 minutes. Post incubation the wells were washed 5 times with diluted wash buffer. 100μl of HRP-Conjugate was then added to each well and the plate was incubated at 37^0^C for 30 minutes. The wells were washed again washed 5 times and 50μl of Chromogen Solution A and then 50μl of Chromogen Solution B was added into each well. The plate was then incubated at 37°C for 15 minutes in dark. 50μl of Stop Solution was added into each well and mixed gently. Absorbance was measured using PR4100 microplate reader, Bio-Rad, USA (dual filter) with reference wavelength at 600~650nm. Cut-off value (C.O.) was calculated as C.O= Nc + 0.16 (Nc = the mean absorbance value for three negative calibrators). The biomedical wastes generated during the process were disposed of according to BMW disposal protocol. The tested serum samples were stored at -80^0^C with proper labelling.

**RESULT**

Supplementary Table 1 shows the participants' household characteristics, while the nature of contact are presented in Supplementary Table 2.

**Supplementary** **Table 1: Household Characteristics of Primary Cases and their Contacts**

|  | **Mean ± SD** | **Median (IQR)** |
| --- | --- | --- |
| **Household Size** | 4·4 ±1·9 | 4 (3,5) |
| **No. Of rooms** | 3·5 ± 1·5 | 3 (2,4) |
| **No. of Bedrooms** | 2·4 ± 0·8 | 2 (2,3) |
| **Overcrowding** | Yes 33 (33·3%) | No 66 (66·7%) |

**Supplementary** **Tables 2: Nature of contact of Household contacts (N=316)**

| **Characteristic** | **Yes (%)** | **No (%)** |
| --- | --- | --- |
| Share Room | 194 (61.4%) | 122 (38.6%) |
| Take Care | 106 (33.5%) | 210 (66·5%) |
| Hug | 33 (10·4%) | 283 (89·6%) |
| Kiss | 15 (4·7%) | 301 (95·3%) |
| Shake Hand | 28 (8·9%) | 288 (91·1%) |
| Share meal | 85 (26.9%) | 231 (73·1%) |
| Eat in the same plate | 14 (4·4%) | 302 (95·6%) |
| Drink in same cup | 14 (4·4%) | 302 (95·6%) |
| Share utensils | 23 (7·3%) | 293 (92·7%) |
| Sleep in same room | 54 (17·1%) | 262 (82·9%) |
| Share Toilet | 110 (34·8%) | 206 (65·2%) |

## **Symptom Profile of Cases and Contacts**

As depicted in Supplementary Figure 2, at least 84.8% of the index cases were symptomatic with the most common symptoms being fever, sore throat, cough, muscle ache and headache. Loss of smell, chills, rhinorrhea, arthralgia and dyspnoea was found in a lower proportion. Other symptoms like rash, vomiting, nausea etc. were rather rare. The symptomatology among the household contacts showed a similar pattern however in a much lower proportion. The most common co-morbidity present among the enrolled participants was found to be diabetes and hypertension (Supplementary Figure 3).


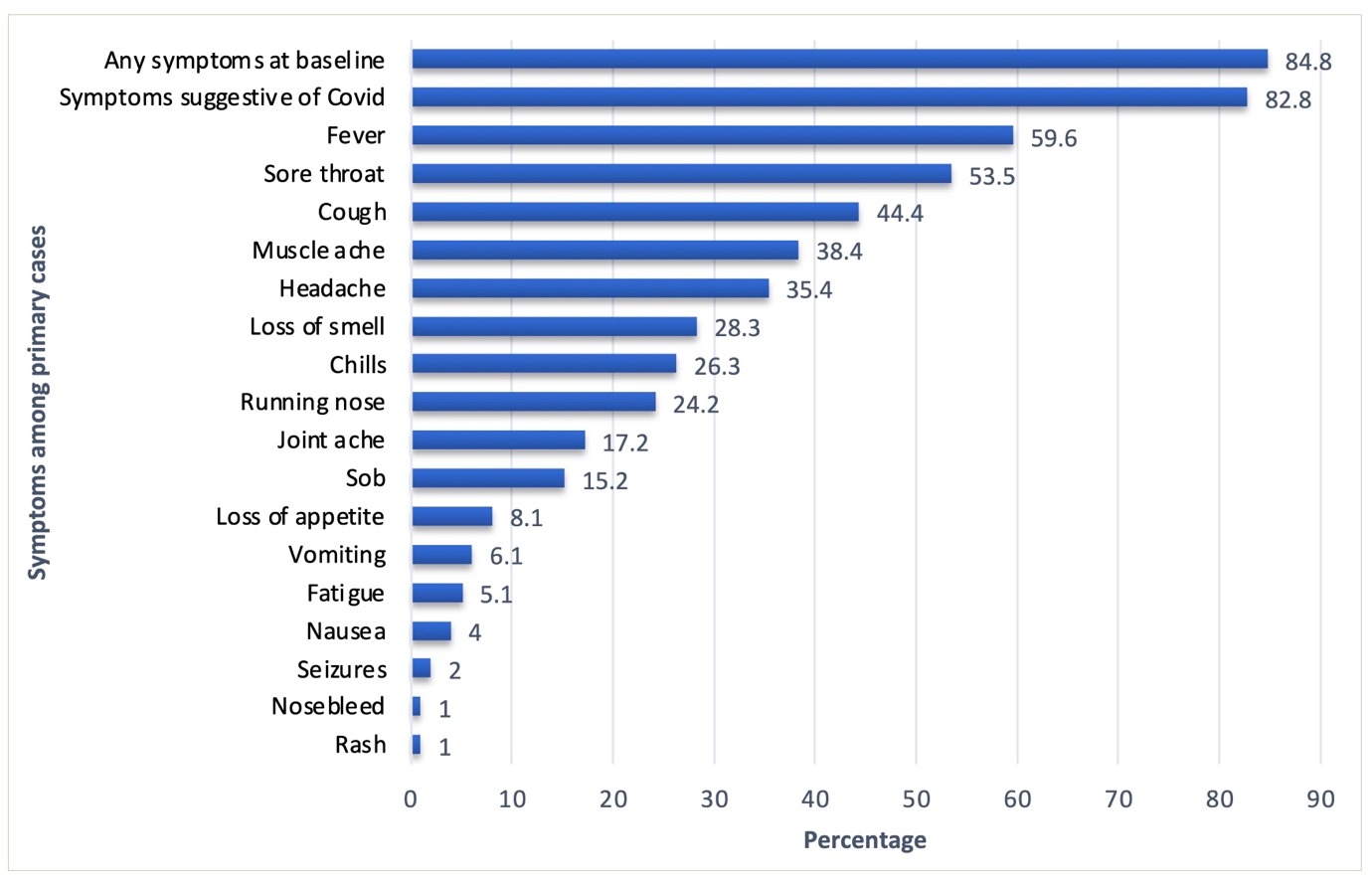


**Supplementary** **Figure 2: Symptom profile of index case**

* Symptoms suggestive of CoVID were Fever, Sore throat, running nose, cough, SOB, Chills, Vomiting, Nausea, Headache, Muscle ache, Loss of appetite, loss of smell, Fatigue


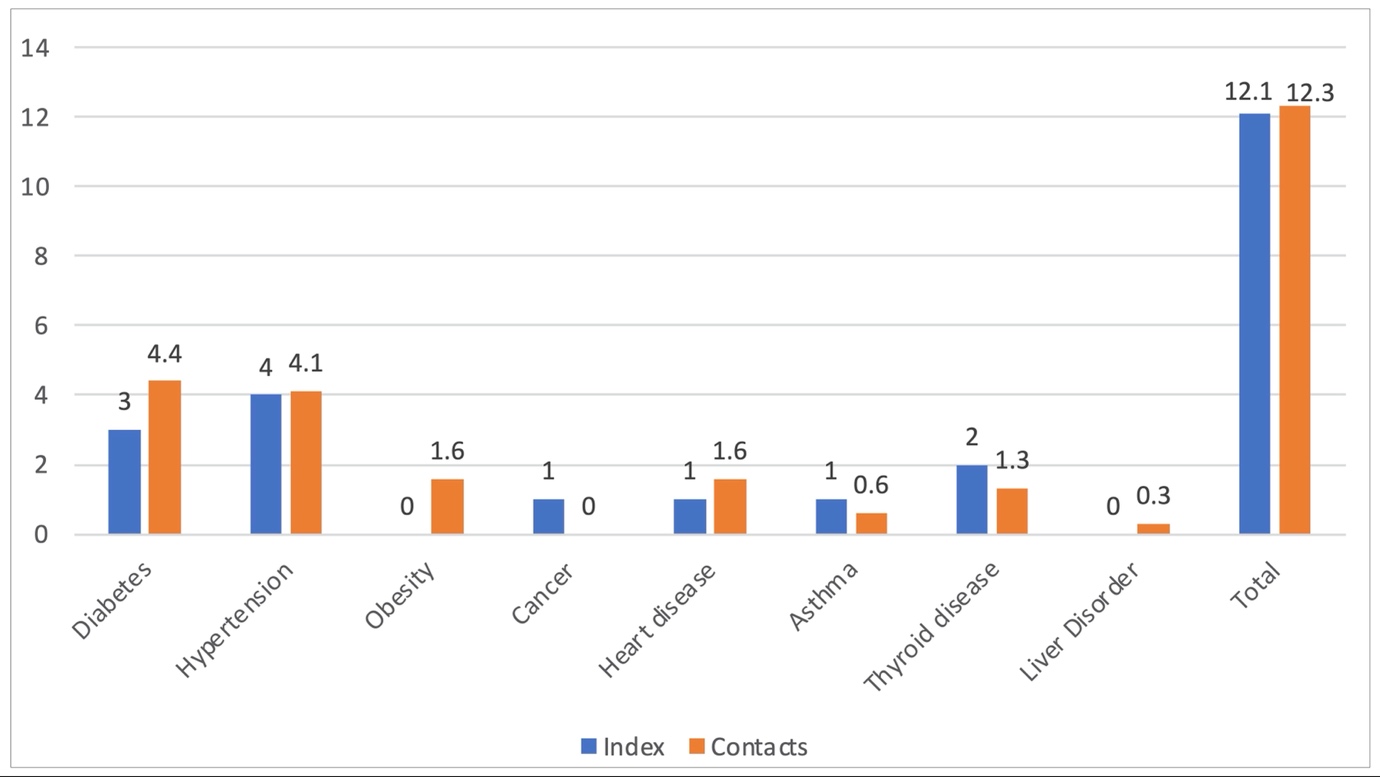


**Supplementary** **Figure 3: Comorbidity Profile of Index case and household contacts**

***** Nil for HIV/ Lung, Hematological, kidney disease/ Neurological impairment/ Immunocompromised /Bone marrow transplantation

**Supplementary** **Table 3: Proportion of symptomatic and asymptomatic Infection**

| **Symptoms during** | **Primary Case (n=99)** | **Household Contacts (N=316)** | **Secondary cases (n=141)** |
| --- | --- | --- | --- |
| **Day 1** | 84 (84.8%) | 94 (29.7%) | 69 (48.9%) |
| **Day 1-7** | 66 (66.7%) | 107 (33.9%) | 77 (54.6%) |
| **Day 7-14** | 41 (41.4%) | 66 (20.9%) | 48 (34.0%) |
| **Day 14-21** | 18 (18.2%) | 23 (7.3%) | 17 (12.1%) |
| **Day 21- 28** | 10 (10.1%) | 15 (4.7%) | 7 (5.0%) |
